# Bile Acids and GPBAR-1: Dynamic Interaction Involving Genes, Environment and Gut Microbiome

**DOI:** 10.3390/nu12123709

**Published:** 2020-11-30

**Authors:** Piero Portincasa, Agostino Di Ciaula, Gabriella Garruti, Mirco Vacca, Maria De Angelis, David Q.-H. Wang

**Affiliations:** 1 Clinica Medica “A. Murri”, Department of Biomedical Sciences & Human Oncology, University of Bari Medical School, 70124 Bari, Italy; agostinodiciaula@tiscali.it; 2 Section of Endocrinology, Department of Emergency and Organ Transplantations, University of Bari “Aldo Moro” Medical School, Piazza G. Cesare 11, 70124 Bari, Italy; gabriella.garruti@uniba.it; 3 Dipartimento di Scienze del Suolo, Della Pianta e Degli Alimenti, Università degli Studi di Bari Aldo Moro, 70124 Bari, Italy; mirco.vacca@uniba.it (M.V.); maria.deangelis@uniba.it (M.D.A.); 4 Department of Medicine and Genetics, Division of Gastroenterology and Liver Diseases, Marion Bessin Liver Research Center, Einstein-Mount Sinai Diabetes Research Center, Albert Einstein College of Medicine, Bronx, NY 10461, USA; david.wang@einsteinmed.org

**Keywords:** bile, cholestasis, FXR, metabolic syndrome, nuclear receptors, TGR5, thermogenesis

## Abstract

Bile acids (BA) are amphiphilic molecules synthesized in the liver from cholesterol. BA undergo continuous enterohepatic recycling through intestinal biotransformation by gut microbiome and reabsorption into the portal tract for uptake by hepatocytes. BA are detergent molecules aiding the digestion and absorption of dietary fat and fat-soluble vitamins, but also act as important signaling molecules via the nuclear receptor, farnesoid X receptor (FXR), and the membrane-associated G protein-coupled bile acid receptor 1 (GPBAR-1) in the distal intestine, liver and extra hepatic tissues. The hydrophilic-hydrophobic balance of the BA pool is finely regulated to prevent BA overload and liver injury. By contrast, hydrophilic BA can be hepatoprotective. The ultimate effects of BA-mediated activation of GPBAR-1 is poorly understood, but this receptor may play a role in protecting the remnant liver and in maintaining biliary homeostasis. In addition, GPBAR-1 acts on pathways involved in inflammation, biliary epithelial barrier permeability, BA pool hydrophobicity, and sinusoidal blood flow. Recent evidence suggests that environmental factors influence GPBAR-1 gene expression. Thus, targeting GPBAR-1 might improve liver protection, facilitating beneficial metabolic effects through primary prevention measures. Here, we discuss the complex pathways linked to BA effects, signaling properties of the GPBAR-1, mechanisms of liver damage, gene-environment interactions, and therapeutic aspects.

## 1. Introduction

Bile acids (BA) are amphipathic molecules made from cholesterol in the liver in the pericentral hepatocytes. BA are conjugated to taurine or glycine to increase their solubility, are actively secreted into the bile canaliculus, and become the major lipid components of bile. During fasting, bile is mostly diverted and stored in the gallbladder, where water is reabsorbed, and bile concentration occurs. During the interprandial phase, a low-grade secretion of bile occurs in the intestine. During the postcibal period, dietary fat in the upper intestine stimulates gallbladder contraction in response to the enterohormone cholecystokinin. This step releases highly concentrated bile into the duodenum. In the intestine, BA promote the emulsification and absorption of dietary fat, i.e., cholesterol, triglycerides, and fat-soluble vitamins. Following ileal and colonic reabsorption, BA undergo continuous enterohepatic circulation several times daily. In the liver and intestine, BA suppress their own synthesis, and recent evidence shows that BA are potent signalling molecules with modulatory effects on epithelial cell proliferation, gene expression, fibrogenesis, as well as lipid and glucose metabolism. Such effects are the consequence of BA acting as endogenous ligands and activation of the nuclear farnesoid X receptor (FXR or NR1H4), the membrane-associated G-protein-coupled bile acid receptor-1 (GPBAR-1, also known as transmembrane G protein-coupled receptor 5, TGR5), and sphingosine-1-phosphate receptor 2 (S1PR2) in the liver, intestine, muscle and brown adipose tissue [1,2,3]. Physiologically, BA are confined almost completely within the enterohepatic circulation, as only traces escape in the general circulation. [4] Integrity of the enterohepatic circulation is therefore central to biliary homeostasis.

This review will discuss in detail the complex pathways underlying BA homeostasis in physiology and during BA overload, their role as signalling molecules in particular for GPBAR-1. We also discuss the multiple effects of this receptor on bile composition, cell lines, inflammation, gene-environment interaction, together with potential therapeutic approaches.

## 2. BA Synthesis, Secretion, Biotransformation, and Absorption

**Figure 1:**
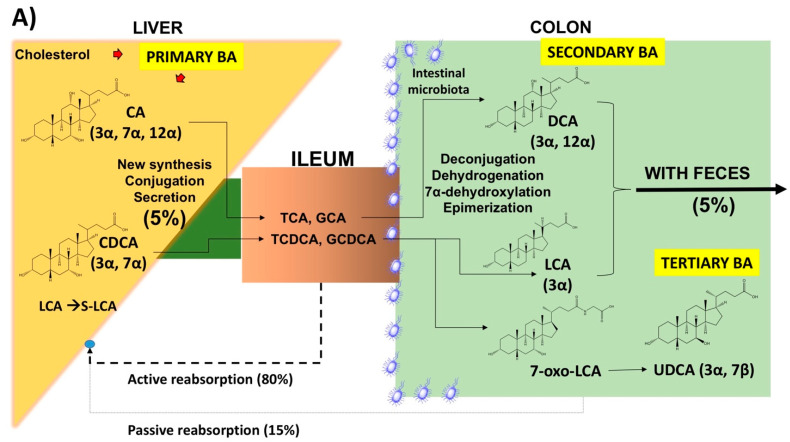

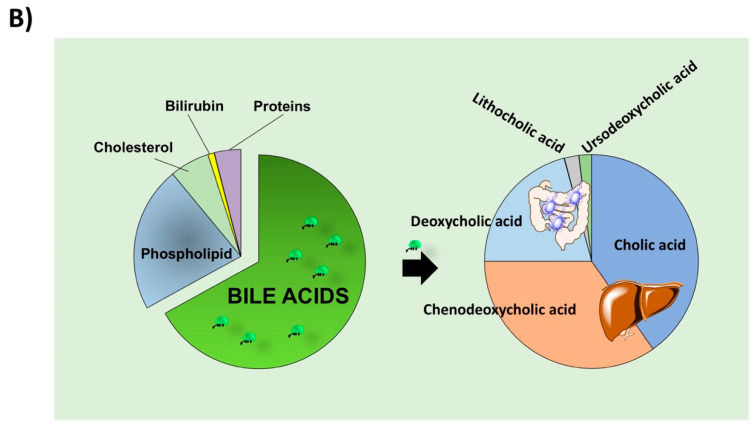
(**A**) Sites of synthesis and metabolism of primary, secondary, and tertiary bile acids (BA) in humans. Cholesterol in the liver undergoes modification of the sterol ring, oxidation, and shortening of the side chain. The classical “neutral” pathway involves the cytochrome P450 enzyme, cholesterol 7α-hydroxylase (CYP7A1), and contributes to about 75–90% of total BA pool consisting of cholic acid (CA) and chenodeoxycholic acid (CDCA). The alternative “acidic” pathway is mitochondrial and contributes to 10–25% of total BA pool [1,7] with the rate-limiting enzyme cholesterol 27α-hydroxylase (CYP27A1) and then 25-hydroxycholesterol 7-alpha-hydroxylase (CYP7B1) [8,9] to produce CDCA. BA in the liver undergo conjugation with amino acids, glycine or taurine (ratio of 3:1), via N-acyl amidation at carbon 24 of the aliphatic side chain [10] and active biliary secretion. In the colon the resident bacteria interact with primary BA by dehydroxylation, dehydrogenation, 7α-dehydroxylation and epimerization. By this pathway, secondary BA are the dihydroxy deoxycholic acid (DCA) and the monohydroxy lithocholic acid (LCA). The 7α-dehydrogenation of CDCA forms the dihydroxy 7α-oxo-LCA which is metabolized to the “tertiary” 7β-epimer, the dihydroxy ursodeoxycholic acid (UDCA). In the liver, a small amount of LCA is quickly transformed to sulphonated form (S-LCA). In the terminal ileum, BA uptake is about 80% by active transport within the enterocytes. In the colon, secondary BA undergo passive diffusion and reabsorption (~15%) [11] into the portal tract. (**B**) Relative composition of hepatic and gallbladder bile in healthy humans. Left: major solutes; right: contribution of individual bile acids. Changing the composition of the BA pool could ultimately influence the signalling ability of BA on nuclear receptors such as farnesoid X receptor (FXR), pregnane X receptor (PXR), vitamin D receptor (VDR), as well as the membrane-associated receptors G protein-coupled bile acid receptor 1 (GPBAR-1) and sphingosine-1-phosphate receptor 2 (S1PR2) [12,13,14].

The complex pathways leading to BA homeostasis in liver and intestine [1,5,6] are summarized in Figure 1. 

**Figure 2:**
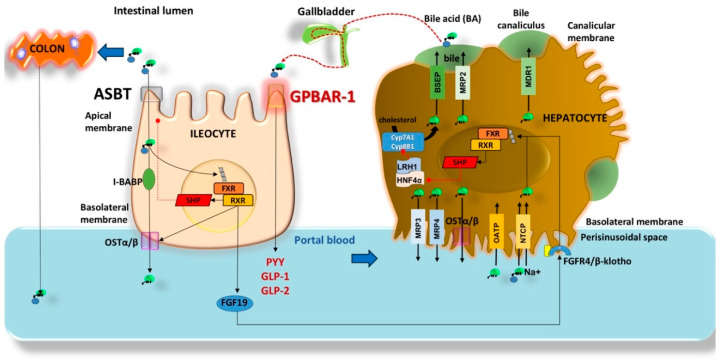
Mechanisms governing bile acid (BA) enterohepatic circulation. In the liver BA undergo secretion into the perisinusoidal space by multidrug resistance proteins (MRP3, MRP4), and heterodimeric organic solute transporter (OSTα/β). Uptake is via organic anion transporting polypeptides (OATP) and Na+−taurocholate cotransporting polypeptide (NCTP). In the bile canaliculus, BA are secreted by bile acid export pump (BSEP) [21], MRP2, and multidrug resistance protein 1 (MDR1), and stored in the gallbladder upon neurohormonal-mediated contraction. In the terminal ileum, BA uptake occurs across the apical sodium dependent bile acid transporter (ASBT), the intracellular transport requires the intestinal BA binding protein (I-BABP); the secretion into the portal vein requires OSTα/β. BA signal the nuclear receptor Farnesoid X receptor (FXR) and retinoid X receptor (RXR) with effects on the small heterodimer partner (SHP), OSTα/β, and synthesis of the human enterokine fibroblast growth factor 19 (FGF19). BA also signal the ileal membrane receptor GPBAR-1 governing the secretion of peptide YY (PYY), glucagon-like peptide 1 (GLP-1) and glucagon-like peptide 2 (GLP-2) with metabolic effects (see text) [9,22]. Reabsorbed BA undergo peripheral spill over into the systemic circulation by about 10–50% [23]. About 15% of BA enter the colon for biotransformation into secondary BA and passive reabsorption. BA re-entering the liver can interact with GPBAR-1 in Kupffer cells and FXR-RXR-SHP (hepatocytes) [24] pathway which inhibits the activity of hepatocyte nuclear factor 4 (HNF4α) and liver-related homologue-1 (LRH1), resulting in inhibited expression of target genes governing BA synthesis (*CYP7A1* and *CYP8B1*) and fatty acid synthesis. At low concentrations of BA, however, LRH-1 acts with LXR to trigger BA synthesis [25,26,27]. The circulating BA undergo renal uptake by the ASBT in the proximal tubules. MRP 2, 3, 4 transporters regulate glomerular filtration of BA [28]. The dashed red lines (-----●) indicate inhibition.

The key events contributing to the enterohepatic circulation of BA appear in Figure 2 [1,15,16,17]. According to this scenario, most BA in the pool stay in the enterohepatic circulation due to the following essential steps: primary BA synthesis (0.2–0.6 g/day), formation of the total (primary and secondary) BA pool (~3 g, mainly in the liver and intestine), active (80%) BA reabsorption at the terminal ileum and passive diffusion (15%) in the colon, daily recirculation within the axis which includes liver secretion, intestinal flow and reabsorption, portal blood flow, liver uptake (4–12 cycles per day), and dynamic increase of the BA pool by 4–12 folds (=12–36 g/day). About 5% (i.e., 0.2–0.6 g per day) of the secreted BA are lost in faeces, equalling the amount of hepatic synthesis (0.2–0.6 g/day) [5,9], urinary excretion of BA is minimal (<1 µM/day), and fasting serum BA concentration in healthy subjects is 0.2–0.7 μM and increases to 4–5 μM after each meal [5,18,19,20].

## 3. BA as Signalling Molecules

BA act as signaling molecules mainly acting on the FXR, and the membrane-associated receptors, GPBAR-1, and sphingosine 1 phosphate receptor 2 (S1PR2). These targets generate different effects (Table 1).

### 3.1. GPBAR-1

GPBAR-1 (previously known as Takeda G protein coupled receptor, TGR5) [62] is expressed in the enteroendocrine L-cells of the intestine [71], Kupffer cells (not on the hepatocyte plasma membrane) [45,72,73,74], cholangiocytes, gallbladder, brown adipose tissue, skeletal muscle, macrophages, and monocytes [9,13,51,75,76,77,78]. In rodent hepatocytes, GPBAR-1 is weakly present, while its expression is evident in the gallbladder, the biliary tract, endothelial cells, and Kupffer cells [51]. In mice, the gallbladder has the highest expression of GPBAR-1 [79]. Secondary conjugated (taurine > glycine) BA, DCA and LCA, are the most potent endogenous ligands (EC50: 0.5–1 µM) [2,76,80]. Primary BA, CA and CDCA, have less affinity (five- to tenfold lower). The tertiary BA (UDCA) has the lowest affinity for GPBAR-1 (EC50: 36 µM) [76,81]. GPBAR-1 acts also as neurosteroid receptor.

**Figure 3:**
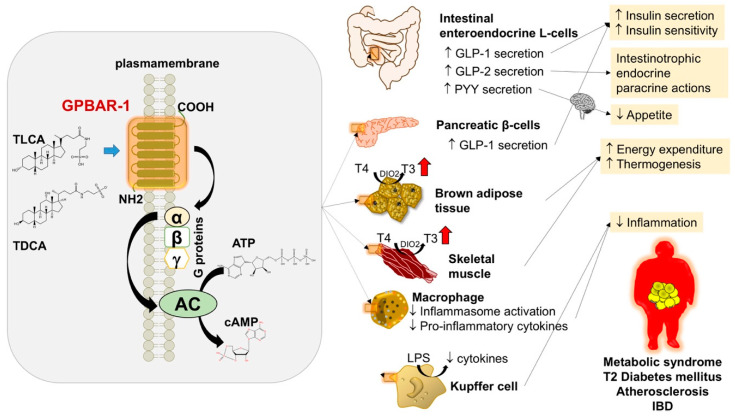
Effects of BA signalling through activation of the GPBAR-1 in target tissues [2,96]. The example depicts the action of the two most potent BA acting at the level of the receptor. The rank order of potency is TLCA > TDCA > TCDCA > TCA [9]. Binding to GPBAR-1 activates a stimulatory Gα protein which triggers adenylate cyclase (AC) activation and cyclic AMP (cAMP) production. In the intestine, GPBAR-1 activation increases the secretion of the incretin GLP-1 (intestine, β), which drives metabolic effects on glucose homeostasis [97]. Increased secretion of GLP-2 induces potent intestinotrophic endocrine/paracrine effects, i.e., increased intestinal mucosal growth, enhanced activity of several brush border enzymes, delay of gastric emptying, and increased absorption of nutrients. Increased secretion of PYY brings anorexigenic effects (appetite reduction) [98]. Ultimate beneficial effects may be on metabolic syndrome, T2 diabetes mellitus, atherosclerosis, and inflammatory bowel diseases. Abbreviations: cAMP, cyclic adenosine monophosphate; ATP, adenosine triphosphate; DIO2, type II iodothyronine deiodinase; GLP-1, glucagon-like peptide 1; GLP-2, glucagon-like peptide 2; GPBAR-1, G-coupled bile acid receptor 1; IBD, inflammatory bowel disease; LPS, lipopolysaccharide; PYY, peptide YY; T3, active thyroid hormone; T4, inactive thyroxine; TDCA, taurodeoxycholic acid.

Activation of the GPBAR-1 receptor leads to cAMP production and possibly calcium mobilization [82,83], and to downstream activation of cAMP-response element (CRE)-binding proteins (CREBs) in target cells (Figure 3).

The effects of GPBAR-1 are evident in the liver [84,85] and extraintestinal tissues [56,86,87,88], and contribute to hepatoprotection, as well as to metabolic regulation [3,45,46,47]. BA-mediated stimulation of GPBAR-1 in the intestine is involved in GLP-1, GLP-2, and PYY-mediated effects with increased insulin secretion and/or sensitivity [43,44], intestinotrophic paracrine actions, and decreased appetite, respectively [2]. Insulin secretion and/or sensitivity results also from GPBAR-1 mediated release of GLP-1 from pancreatic β-cells [89,90].

The regulation of energy homeostasis [1,48,49,50] relies on increased host energy expenditure [43,56,91,92,93] in skeletal muscle and brown adipose tissue, where GPBAR-1 stimulation results in local activation of the type II iodothyronine deiodinase (DIO2). This step, in turn, transforms the inactive thyroxine (T4) to active thyroid hormone, 3, 5, 3′-triiodothyronine—T3, a key regulator of metabolism and energy homeostasis. Experimental data support the metabolic relevance of GPBAR-1, since the synthetic agonist INT-777 is effective in adipose tissue by stimulating mitochondrial biogenesis and fission, while increasing overall adipocyte mitochondrial content and mitochondrial (uncoupled) respiration capacity of white adipose tissue [94]. GPBAR-1 KO mice on a high-fat diet exhibited significant fat accumulation with body weight gain compared with that of the wild-type mice [79], while in mice, the activation of GPBAR-1 by INT-777 stimulates the release of the incretin GLP-1, protecting from diabetes and obesity [43].

Some metabolic effects evident after bariatric surgery [49], and cholecystectomy [48,50] might also involve the rearrangement of the enterohepatic circulation of BA and their effects on GPBAR-1.

In immune cells (macrophages and Kupffer cells), GPBAR-1 has immunosuppressive effects by decreasing proinflammatory cytokine expression. The mechanism involves the inhibition of nuclear translocation of NFκB in a cAMP-PKA dependent manner [56,57]. A direct inhibition of the NLRP3 inflammasome by GPBAR-1 agonist INT-777 is another anti-inflammatory mechanism [61]. This GPBAR-1 mediated effect, however, should be considered with caution, due to the proinflammatory potential of BA in the liver [95].

#### 3.1.1. Effect of GPBAR-1 on Bile Composition

GPBAR-1 has potentials to influence bile composition. Although mechanisms remain largely unknown [99,100,101], they involve BA synthesis, intestinal biotransformation and uptake, the biliary tract, and kidneys (Table 2).

GPBAR-1 is absent or weakly expressed in hepatocytes, but highly expressed in Kupffer cells, cholangiocytes and in the gallbladder epithelial cells. In line with this, GPBAR-1 effects are mild on BA synthesis and canalicular secretion. GPBAR-1-KO mice exhibit a smaller BA pool size (up to 25%) [79], more hydrophobic composition and a mildly decreased bile flow. GPBAR-1 agonist treatment has uncertain effects on bile flow [99,100]. GPBAR-1 stimulation has a BA-independent choleretic effect in the isolated perfused rat liver [106]. Furthermore, mRNA expression of the enzymes involved in BA synthesis and BA transporters is similar between wild-type and GPBAR-1 KO mice [47]. By contrast, GPBAR-1 activation has a stronger effect on ductular (cholangiocyte-dependent) component of bile secretion, i.e., on the regulation of CFTR-dependent Cl- secretion in human gallbladder [104] and other epithelial cells [107,108], as well as on biliary pH regulation (dependent on biliary HCO3- and Cl- secretion) after partial hepatectomy in mice [47]. GPBAR-1 regulates the function of cholangiocyte cilia [109,110] which contribute to biliary HCO3- secretion [111].

As seen in kidney epithelial cells [108], GPBAR-1 might also contribute to regulation of water reabsorption in the biliary tract. Ultimately, by acting on biliary chloride and bicarbonate transport GPBAR-1 might contribute to decrease BA protonation and toxicity on the hepatocytes and cholangiocytes [99,112]. GPBAR-1 KO mice display more hydrophobic BA pool in bile, plasma, liver and faeces, compared with wild-type mice [47,100,101]. In this context the amount of muricholic acid and muricholic acid/CA ratio decrease, leading to increased secondary BA. Of note, sequestering BA with cholestyramine treatment, as well as Kupffer cell depletion, improve the postpartial hepatectomy phenotype of GPBAR-1 KO mice, reducing severe liver injury and impaired BA urinary elimination in a scenario of BA overload. Again, the study points to a role of GPBAR-1 in liver protection acting on bile hydrophobicity and cytokine secretion [47]. A further beneficial effect from BA sequestering with cholestyramine can be the decreased FXR activation and, in turn, an increased CYP7A1 activity, leading to higher BA synthesis and reducing cholesterol levels, finally resulting in a decreased liver injury [113].

#### 3.1.2. Various Effects on Cell Lines

**Figure 4:**
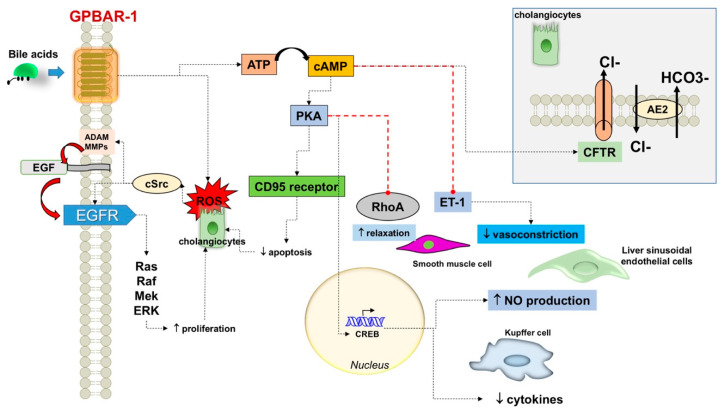
Additional effects of G protein-coupled bile acid receptor 1 (GPBAR-1) stimulation. Abbreviations: ↑, increased; ↓, decreased; AE2, anion exchanger 2; ATP, adenosine triphosphate; cAMP, cyclic AMP; CREB, cAMP-responsive element-binding protein; cystic fibrosis transmembrane conductance regulator; EGF, epidermal growth factor; ET-1, endothelin 1; MMP, matrix-metalloproteinase; NO, nitric oxide; PKA, protein kinase A; ROS, reactive oxygen species.

Several tissues express GPBAR-1 and additional effects are anticipated (Table 3). Following GPBAR-1 stimulation, a cascade of events involves stimulatory and inhibitory signaling pathways in different cells, i.e., cholangiocytes (see also above), smooth muscle cells, macrophages (Kupffer cells), and liver sinusoidal endothelial cells. In particular, Figure 4 describes additional effects of GPBAR-1 stimulation and how GPBAR-1 activation leads to cyclic AMP (cAMP) production with outcomes at four cellular levels. Activation of protein kinase A (PKA) results in phosphorylation of the CD95 receptor decreases apoptosis (cholangiocytes); the nuclear activation of the cAMP-responsive element-binding protein (CREB) results in decreased release of cytokines (Kupffer cells) and production of nitric oxide (liver sinusoidal endothelial cells, which are component of the reticuloendothelial system). PKA inhibits RhoA in smooth muscle cells and starts relaxation. The GPBAR-1-cAMP-dependent inhibition of endothelin-1 will further decrease the vasoconstriction of liver sinusoidal endothelial cells. Another effect of the GPBAR-1-cAMP interaction is the activation of the cystic fibrosis transmembrane conductance regulator (CFTR) in cholangiocytes which governs chloride (Cl-) secretion and anion exchanger 2 (AE2)-mediated bicarbonate (HCO3-) transport across the apical membrane. GPBAR-1 activation in cholangiocytes triggers the production of reactive oxygen species. This step, in turns, activates Src kinase with release of epidermal growth factor (EGF) involving a matrix-metalloproteinase-dependent pathway. EGF-dependent transactivation of its receptor (EGFR) induces the Ras/Raf/Mek/ERK cascade which promotes cell proliferation.

GPBAR-1 might contribute to the release of nitric oxide from the endothelium [51,52,53]. GPBAR-1 could modulate portal blood flow via release of vasodilators such as hydrogen sulphide (H2S), and inhibition of ET-1 transcription [54,55].

## 4. Mechanisms of Damage

### 4.1. BA Overload

The molecular amphipathic structure of BA makes the pool either protective or toxic depending on the tight maintenance of the hydrophilic-hydrophobic balance. This balance results from the continuous enterohepatic circulation of synthesis-secretion (liver), absorption (intestine), uptake (liver), and resecretion (liver) during the process of enterohepatic circulation, with minimal faecal loss and the intestinal biotransformation. The interaction between BA and the main BA nuclear receptor, FXR, in the distal small intestine is essential in this respect (see below). Any step interfering with BA homeostasis might produce clinically relevant conditions. For example, defective ileal BA transport is associated with faecal BA loss. Excess of BA retention, by contrast, could lead to hepatic damage via cholestasis, hepatic steatosis, fibrosis and liver tumour [1,114].

**Figure 5:**
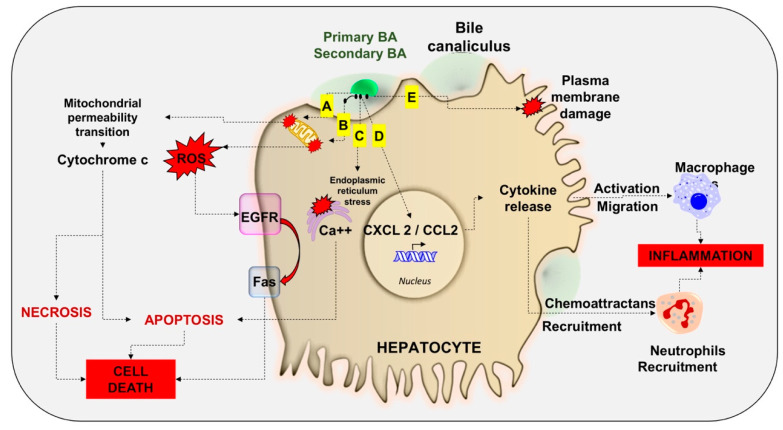
The cascade of damage in the hepatocytes, as triggered by excess of primary and/or secondary BA. (**A**) Mitochondrial damage and release of reactive oxygen species (ROS), with activation of epidermal growth factor receptor (EGFR) and Fas-death receptor pathway. (**B**) Endoplasmic reticulum stress with cytosolic Ca++ release contributing to apoptosis. (**C**) Induction of chemokine (C-X-C motif) ligand 2 (CXCL2) and chemokine (C-C motif) ligand 2 (CCL2), which act as chemoattractants of neutrophils and activator agents of macrophages. (**D**) Damage at the level of hepatocyte plasma membranes. (**E**) Damage at the level of hepatocyte plasma membranes.

Excess of circulating BA or increased retention of BA may lead to “BA overload”. BA overload could develop at a hepatic and/or systemic level [4] if trans-hepatocyte BA flow becomes insufficient due to reduced sinusoidal and/or canalicular BA transport, or if bile duct obstruction develops. Partial hepatectomy or liver injury are predisposing condition to BA overload [115,116], due to imbalance between the enterohepatic return of BA from the intestine and the hepatic uptake capacity. [3,117]. A feature in this case is a significant BA spillover into the systemic circulation. Both animal models and humans exist in this respect, i.e., after partial hepatectomy in mice and rats [47,117,118], portal vein embolization in rabbits [119], and after CCl4 intoxication in mice [116]. In humans, BA overload occur following partial hepatectomy [117,120] and portal vein embolization [120]. Mechanisms of BA-dependent damage [121,122] may involve mitochondrial damage [123,124,125,126,127] leading to release of cytochrome c, an production of reactive oxygen species (ROS) [128], plasma membrane damage, necrosis, apoptosis and cell death [129]. BA-dependent damage might also be indirect, because of inflammatory processes resulting in cytokine release, recruitment of neutrophils, and activation of macrophages [121,130]. Figure 5 displays how excess BA (either primary or secondary), depending on the hydrophilic-hydrophobic balance, trigger the cascade of damage in the hepatocytes, including mitochondrial membrane damage (mitochondrial permeability transition) releases cytochrome c, and leads to necrosis, apoptosis, and hepatocyte cell death.

Still, BA have a double signalling function leading to either protection (i.e., proliferation in hepatocytes) or toxicity [3]. This is a feature occurring, for example, in the case of extended (>70%) partial hepatectomy or massive hepatocyte loss [3,115,116,131,132,133,134].

FXR-dependent adaptive responses are involved in liver protection after injury and in ameliorating regenerative responses. In fact, basolateral and canalicular BA transporters and enzymes governing BA synthesis and conjugation are also involved [30,47] and contribute to counteract BA overload in the hepatocytes [31]. Hepatocyte cell cycle progression may be modulated by FXR-dependent mechanisms [115], a step contributing to BA homeostasis [135], alcohol-related liver injury [136], and liver regeneration after partial hepatectomy [137,138]. FXR pathways also involve cholangiocyte cell cycle progression [138,139] during BA synthesis suppression [95,140]. BA may cause a proinflammatory effect via activation of the inflammasome, but FXR exhibits anti-inflammatory effects, because of the interaction with NLRP3 proteins [141]. Evidence in both animal models and human disease points to the concept that liver homeostasis depends on tight maintenance of bile hydrophilic-hydrophobic balance [142,143]. Mice developing impaired pathways which involve SIRT1 [144], FGF receptors [145,146], small heterodimer partner (SHP) [147], fibroblast growth factor 15 (FGF15) [148], or put on LCA -enriched diet [149], exhibit an hydrophobic BA pool which interferes with liver repairment. Mouse bile is more hydrophilic than human bile. Mice have a very hydrophilic bile, since the enzyme CYP2c70 (missing in humans) converts CDCA to the more hydrophilic muricholic acid [150]. As expected, Cyp2c70^−^/^−^ mice develop a more human-like hydrophobic BA pool, develop liver inflammation [151] and altered FXR signalling [152].

By contrast, BSEP/abcb11^−^/^−^ mice develop a nonprogressive mild cholestasis [149], likely due to a BA pool enriched in hyper-hydroxylated, less hydrophobic, and less cytotoxic BA [153]. In the human context, progressive familial intrahepatic cholestasis (PFIC) is an autosomal recessive disease causing 15% of cases of neonatal cholestasis. The PFIC2 form has a mutation in the *ABCB 11* gene encoding BSEP protein. These patients have disrupted BA secretion from the hepatocytes, and develop progressive liver fibrosis, cirrhosis and end stage liver disease requiring liver transplantation [154]. Thus, increasing the hydrophilic profile of the BA pool, might be beneficial during cholestatic liver injury in the animal model [155] and in PFIC children (i.e., by increasing the content of tetrahydroxy BA) [156].

### 4.2. Biliary Epithelial Barrier

Maintenance of cholangiocyte paracellular permeability is essential to prevent BA-mediated damage of the hepatocytes during continuous BA flow into the biliary tract and cholestasis. This protection requires the integrity of the so-called blood-biliary barrier [157] involving the biliary epithelium, a proper regeneration and preserved barrier function [158]. In the blood-biliary barrier system, the bile is separated from blood at the level of both hepatocytes and cholangiocytes and tight junctions (TJ), structures consisting of plasma membrane and cytoplasmic proteins [159]. In particular, TJ includes transmembrane proteins (i.e., occludin, claudins and junctional adhesion molecules—JAMs), and cytoplasmic proteins (zonula occludins proteins, ZO) connecting the transmembrane proteins with the actin cytoskeleton [160]. The extracellular domains of TJ aggregate between adjacent cells, seal plasma membranes, and are involved in paracellular permeability. Phosphorylation of TJ transmembrane proteins (i.e., occludin, JAM-A and claudin 4) likely influences the paracellular permeability in different epithelial cells [161,162]. TJ distinctively regulate the function of the diffusion barrier. TJ also govern the selective paracellular exchanges of ions and other small molecules between apical and basolateral sites of cholangiocytes [160].

The assembly of TJ protein is different in hepatocytes and cholangiocytes [163]. While hepatocyte TJ proteins play a role during bile secretion [164,165,166], less is known about TJ in the biliary epithelium [167,168]. Changes of cholangiocyte permeability may lead to leaky bile ducts, and BA-induced liver damage. This is the case with mutations in the TJ associated protein ZO-2 gene [169], as well as mutational defect of claudin-1 [170,171]. In addition, TJ alterations occur during cholestatic diseases [167,168,172], while cytokine and lipopolysaccharide production increases the local permeability [172]. The KO mouse model of γ- and β- catenins develops severe cholestatic liver injury, with changes due to TJ protein expression [173]. Indeed, BA-dependent signalling pathways influence paracellular permeability of intestinal [174] and respiratory epithelial cells [175]. By contrast, BA-induced activation of the GPBAR-1 receptor restores the intestinal and endothelial barriers in mice [52,176].

GPBAR-1 might counteract the damage induced by bile leakage by partly influencing the tight junction protein JAM-A. During BA overload in cholestasis, BA promoted PKCζ-dependent phosphorylation of JAM-A Ser285 via GPBAR-1 activation. In parallel, paracellular permeability decreased, and hepatoprotection occurred with increased transepithelial resistance and reduced paracellular permeability for dextran. In addition, GPBAR-1 KO, as compared with WT mice showed increased dextran diffusion after gallbladder injection and decreased on TGR5 stimulation [78].

### 4.3. Inflammation

Impaired BA homeostasis paves the way to BA-induced liver injury and BA-mediated proinflammatory effects [95,121,130,141]. During cholestasis and sepsis, CDCA and DCA activate NLRP3 inflammasome [177]. CDCA transactivates GPBAR-1-dependent EGFR, and stimulates NLRP3 as well [178]. LCA appears to inhibit NLRP3 activity via a GPBAR-1-cAMP-PKA dependent pathway, phosphorylation and ubiquitination of NLRP3 proteins [61] suggesting that GPBAR-1 signaling prevents NLRP3 inflammasome activation through the disruption of NLRP3-mediated ASC nucleation [179]. Since GPBAR-1 is absent in hepatocytes, other cell lines likely mediate the inflammatory changes i.e., during cholestasis. Neutrophils and related cytokines could initiate the proinflammatory events [141]. In line with this possibility, GPBAR-1 KO mice exposed to partial hepatectomy exhibited increased cytokines, delayed liver regeneration, increased cholestasis [180], and hepatocyte necrosis [47]. The anti-inflammatory effects of GPBAR-1 imply decreased inflammatory immune responses [96,176,181,182]. In Kupffer cells and macrophages, GPBAR-1 activation blunts the production of LPS-induced cytokine [96], and is involved in anti-inflammatory effects [56,77,84,85,86,87,88,183]. For example, GPBAR-1 KO mice in a LPS-induced inflammation model show more severe liver necroses and inflammation compared with wild-type mice. However, activation of GPBAR-1 by 23(S)-mCDCA, a new synthetic, highly selective agonist ligand inhibited the expression of inflammatory mediators in response to nuclear factor NF-κB activation in wild type but not GPBAR-1 KO mouse liver [85]. Excess BA might contribute to inflammatory changes, as well. CDCA is the major hydrophobic primary BA contributing to cholestatic liver injury. CDCA dose-dependently induced NLRP3 inflammasome activation and secretion of proinflammatory cytokine-IL-1β in mice macrophages. Mechanism implies ROS production and K+ efflux partly via GPBAR-1/EGFR downstream signalling (protein kinase B, extracellular regulated protein kinases and c-Jun N-terminal kinase pathways). CDCA is effective in inducing ATP release from macrophages with K+ efflux via P2 × 7 receptor. Another study suggests that GPBAR-1 is a potential target for the treatment of NLRP3 inflammasome-related diseases. Some pathways participating in this scenario appear in Table 4.

Abbreviations: cAMP, cyclic adenosine monophosphate; AKT, protein kinase B; CDCA, chenodeoxycholic acid; CEBP-β, CCAAT/enhancer-binding protein beta; EGFR, endothelial growth factor receptor; LPS, lipopolysaccharide; NF-κB, nuclear factor κB; NLRP3, NLR Family Pyrin Domain Containing 3; mTOR, mammalian target of rapamycin; PKA, protein kinase A.

The role of GPBAR-1 in tumorigenesis requires further studies. GPBAR-1 activation, i.e., by DCA treatment, transactivates EGFR–STAT3 signalling, which plays an important role in cancer progression [185]. BA increase cell proliferation via activation of GPBAR-1and G(q)alpha and Galpha(i-3) proteins in gastric adenocarcinoma [186]. GPBAR-1 activation might influence other cancer lines [187,188,189]. Under certain conditions, production of reactive oxygen species (ROS) might increase [82,186,190], together with cell proliferation and apoptosis in cancer cell lines and tumours [58,59,60].

## 5. Gene-Environment Interactions Involving GPBAR-1

Environmental factors (i.e., dietary habits, lifestyle, maternal dietary factors, air pollution, and ingestion of contaminated food or water) play a critical role in the onset and progression of diseases. In this context, the epigenome acts as an interface between the environment and the genome, modulating gene expression according to environmental exposures [191,192]. Epigenetic implications play also a role in case of metabolic diseases and hormonal homeostasis [191,193]. There are preliminary findings pointing to an involvement of GPBAR-1 (Table 5).

Animal studies found epigenetic effects of early exposure (i.e., in utero, during lactation, in prepuberal age) to chemicals of environmental origin (mainly flame retardants, bisphenols) on gene expression of GPBAR-1. Flame retardants are commonly employed in a number of consumer products (electronic devices, automotive products, foam-based packaging materials, textiles, paint products, carpet padding, and adhesives). Brominated flame retardants including polybrominated diphenyl ethers (PBDEs) and hexabromocyclododecane (HBCDD) may be introduced through inhalation, dermal contact or by ingestion of contaminated food, in particular, high-fat foods and fish, and water. PBDEs may be also transmitted through breast milk [192]. In an animal model of in utero and lactational exposure to PBDEs and HBCDD, dietary intake of these chemicals generated, in the offspring, an increased ovarian gene expression of GPBAR-1 mRNA, showing transgenerational effects of the exposure [194].

Bisphenols are widely diffused food contaminants, usually employed in the manufacture of polycarbonate plastic and epoxy resins. These chemicals, in particular Bisphenol A (BPA), contaminate food and beverages leaching out from containers. The body concentrations of BPA have been specifically linked with obesity, weight gain, and type 2 diabetes [192]. An early postnatal dietary exposure to BPA induced, in mammary tissues from adult Sprague-Dawley rats, epigenetic effects (altered DNA methylation) which significantly affected the expression of a number of genes, including the GPBAR-1 gene. In this case, a decreased methylation is shown. This experimental model confirms the possibility of effects which are secondary to early exposure but still evident in the long term [195]. In vitro, in a cultured model of rat seminiferous tubule, the exposure to BPA at low concentration (1 nM and 10 nM) in the culture medium alters gene expression inducing an upregulation of the GPBAR-1 gene, which is dependent on time of exposure, but independent of BPA concentration [196]. As a consequence of growing evidence pointing to the endocrine disrupting effect of BPA, this chemical has been widely substituted with the analogues Bisphenol F (BPF) and Bisphenol S (BPS). However, preliminary results from experimental studies also raise concerns for the safety of these two analogues [192]. In a model of cultured human primary adipocyte, in vitro exposure to BPA, BPF and BPS induces a deregulation of a number of genes, including an increased expression of GPBAR-1 mRNA [197]. In humans, the environmental exposure to air pollution has been linked with a number of negative health effects, including metabolic diseases [192]. In a series of nonsmoking workers employed in the trucking industry and exposed to traffic-related air pollution, the exposure to three pollutants (i.e., particulate matter ≤ 2.5 microns in diameter, PM2.5; elemental carbon, EC; and organic carbon, OC) has been linked with altered gene expression using a genome wide gene expression microarray analysis. Results show significant gene-environment interactions, including an increased expression of GPBAR-1, which is proportional to exposure to EC and OC [198].

Evidences suggest that exposure to both endocrine disruptors and air pollutants with obesogenic and dysmetabolic potentials [191,193,199] coexist with upregulation of GPBAR-1 gene and increased expression of GPBAR-1 mRNA. This scenario might sound as contradictory, since GPBAR-1 overexpression should generate beneficial metabolic effects. The discrepancy might be partly explained by the pollutant-generated activation of complex pathways (i.e., altered adipogenesis, liver, pancreatic and neurologic dysfunction, insulin resistance, and gut dysbiosis [199]) which, in turn, promote GPBAR-1 activation as a protective mechanism. A similar mechanism can operate during ongoing oxidative stress and inflammatory response following hepatic ischemia/reperfusion injury in the animal model [200]. In addition, exposure to endocrine disruptors might increase primary and secondary BA levels [201], and the mechanism might also involve altered fecal microbiome and adverse effects on the gut-liver axis [202,203,204].

Further studies are certainly needed to better explore the role of environmental factors modulating GPBAR-1 expression in in the distal intestine and in the liver. Possible results may be of major importance mainly in terms of possible primary prevention measures.

## 6. Therapeutic Aspects

**Figure 6:**
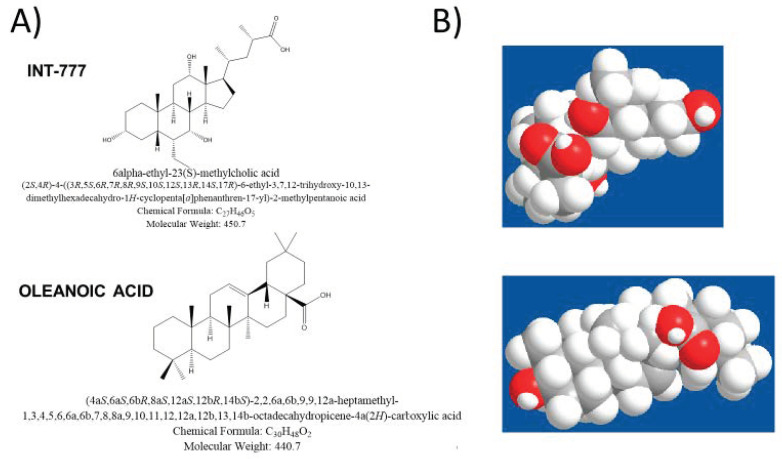
Chemical name, formula, and molecular weight (**A**) and 3D formula (**B**) of the GPBAR-1 agonists INT-777 and oleanoic acid. In the 3D formulas grey = C, red = O, white = H.

Targeting GPBAR-1 could influence aspects related to inflammation and metabolism [2,96]. The potential therapeutic effects derive from the multiple multiorgan effects of GPBAR-1, and the potent GPBAR-1 agonists exist [205]. INT-777 is a potent GPBAR-1 agonist with an EC50 of 0.82 μM [81,205,206]. Another (nonselective) agonist is the triterpenoic oleanoic acid (Figure 6). Some studies test the ability of BA to directly stimulate GPBAR-1. GPBAR-1 modulators are developed based on mouse studies [205], and many studies are experimental and from animal models [2].

### 6.1. Liver Steatosis

In obese mice, INT-777, the GPBAR-1 agonist, effectively decreases liver steatosis [43]. Although definitive clinical studies are missing in this respect, the GPBAR-1 agonists might become an attractive therapeutic option for the treatment of nonalcoholic steatohepatitis (NASH), especially in patients with diabetes and obesity [43,56,91,92]. The GPBAR-1 agonists, INT-767 and INT-777 (effective on FXR as well), decrease hepatic macrovesicular steatosis and protect against ethanol-induced liver injury. The expression of lipogenic genes and the content of hepatic interleukin-1β mRNA expression parallel the augmented ubiquitination of NLRP3 inflammasome. This pathway involves the activation of protein kinase A via cyclic adenosine monophosphate-induced activation [84]. BAR501, a semisynthetic BA derivative, similar to UDCA, is a potent selective agonist of GPBAR-1 and rescues from endothelial dysfunction in rodent models of portal hypertension by exerting genomic and nongenomic effects on cystathione-gamma-liase, eNOS and ET-1 in liver sinusoidal cells [54].

### 6.2. Obesity and Diabetes

GPBAR-1 activation in the macrophages may be a promising approach to preventing insulin resistance and treating type 2 diabetes mellitus and associated inflammatory and metabolic disorders [184]. Few data exist in patients with type 2 diabetes. In a study, the GPBAR-1 agonist SB-756050 is used for six days and the results are disappointing and not consistent, with respect to serum levels of glucose, GLP-1, and PYY [207]. Oleanoic acid, another GPBAR-1 agonist, ameliorates glycemic control in obese mice [208] and decreases plasma triglycerides in patients with hyperlipidemia [209]. UDCA might improve the release of GLP-1 and the efficacy of antidiabetic agent, sitagliptin (Inhibitor of dipeptidyl peptidase 4, DPP-4 inhibitor) (Clinicaltrial.gov NCT01337440), as show in a 12-week trial [210]. More BA might also stimulate GPBAR-1 but the ultimate therapeutic role remains obscure, so far. In addition, other receptors, beside GPBAR-1, might be involved. In healthy humans, TCA infused in the rectum stimulate GLP-1 and PYY secretion [211], while in patients with type 2 diabetes, and healthy control, intragastric infusion of CDCA is associated with increased GLP-1 and glucagon levels [212]. In patients treated by bariatric surgery, both CDCA and UDCA stimulate GLP-1 release [213]. Due to the effect on energy expenditure by GPBAR-1 in brown adipose tissue, BA might play a therapeutic role. This is the case with CA and CDCA feeding, which reverses obesity in the mouse model [93,214]. CDCA increase brown adipose tissue activity in humans likely by increasing whole-body energy expenditure [215].

### 6.3. Atherosclerosis

Researchers have tested the possibility that the GPBAR-1 agonists, by modulating the function of macrophages, might also be effective in preventing the atherosclerotic process [61]. Again, studies are restricted to the animal model. The GPBAR-1 agonist, INT-777, effectively reduces the atherosclerotic process in LDLR KO mice, acting on macrophages via decreased proinflammatory cytokine production and decreased lipid uptake. GPBAR-1 exert an inhibitory effect on the NLRP3 inflammasome [56], thus contributing to decrease the proinflammatory pathway in atherosclerosis [61]. Oleanoic acid, another GPBAR-1 agonist [208], exhibits anti-atherosclerotic effect and hypolipidemic effect in the animal model (mice and rabbits) [209,216]. Studies in humans confirm the lipid-lowering effect of oleanoic acid, but observation time is only one month [217].

### 6.4. Inflammatory Bowel Diseases

The inflammatory process in IBD might be attenuated by GPBAR-1 stimulation. GPBAR-1 KO mice display increased intestinal permeability [176]. In patients with Crohn’s disease, another GPBAR-1 agonist (3-Aryl-4-isoxazolecarboxamide) reduced cytokine production by acting on mononuclear cells isolated from the lamina propria. TNBS and oxazolone induced colitis in mice, and the inflammation was attenuated by BAR501 a small molecule agonist for GPBAR-1. The mechanism likely was an IL-10-dependent phenotype shift of activated macrophages [218]. Similar beneficial effects on chemically-induced colitis were evident with the GPBAR-1 agonists triterpenoids OA and betulinic acid [176,219], BIX02694 [220].

### 6.5. Potential Drawbacks Associated with GPBAR-1 Stimulation

Overall, the anticipated beneficial therapeutic effects associated with GPBAR-1 stimulation, must be balanced with the potential drawbacks shown in both animal and human research (Table 6). Due to the widespread distribution of GPBAR-1, several target organs can be involved, including gallbladder, pancreas, skin, CNS, endothelium, and gastrointestinal epithelia.

### 6.6. Summary of Protective Mechanisms and Action Target Involving GPBAR-1.

**Figure 7:**
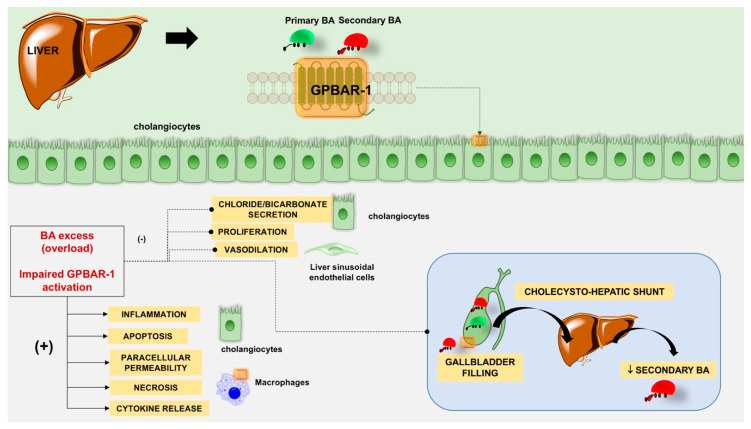
Main mechanisms of hepatoprotection involving GPBAR-1. The signs (−) or (+) indicate inhibition or activation, respectively.

Figure 7 depicts the main mechanisms of hepatoprotection involving GPBAR-1.

The activation of GPBAR-1 in different cells takes part in the integrity of the blood-biliary barrier by promoting healthy cholangiocyte proliferation, paracellular permeability (preserving the function of the tight junctions), and biliary excretion of chloride and bicarbonate. In the gallbladder, GPBAR-1 promotes refilling, the cholecysto-hepatic shunt with reabsorption of BA and decrement of secondary BA. GPBAR-1 activation reduces sinusoidal vasodilatation, hepatic vascular tone and portal pressure. Conditions characterized by excess of BA (overload) and/or impaired GPBAR-1 activation are associated with inhibition or activation of specific mechanisms leading to damage at various cellular levels, and decreased hepatoprotection.

## 7. Conclusions

**Figure 8:**
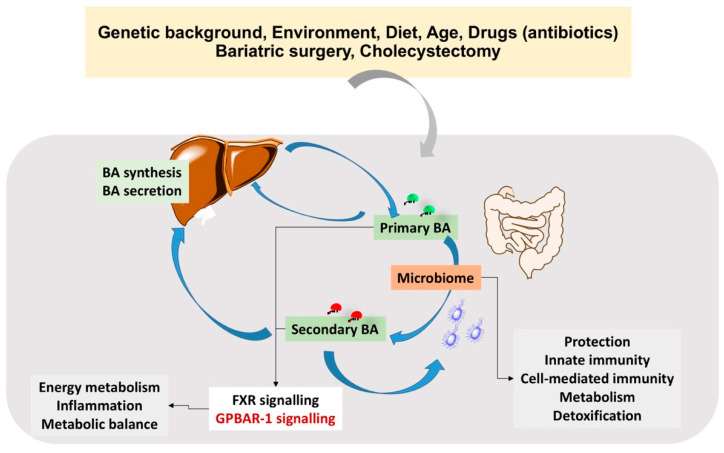
Mutual interaction between BA and gut microbiome. BA undergo biotransformation by the microbiome, a step contributing to the final composition of the BA pool. BA play an important role as signalling molecules (FXR and GPBAR-1), contributing to several metabolic functions and with a role in immunological and inflammatory aspects. BA also control gut bacteria growth.

BA act as cholesterol carriers in bile, play a role in the digestion and absorption of nutrients, and display protecting or damaging properties on the liver, depending on their structure, and on the hydrophilic-hydrophobic balance of the BA pool. The gut microbiome governs BA pool composition, and related immunological, and metabolic functions (Figure 8).

In addition, BA are potent signalling messengers acting on FXR, and the membrane-associated receptor, GPBAR-1. These receptors are at the crossroads of pathways able to prevent the consequences of BA overload. In particular, novel evidence points to the relationship between BA and GPBAR-1 with respect to metabolism, thermogenesis, inflammation, cholangiocyte secretion, biliary epithelial barrier permeability, sinusoidal blood flow, enterohepatic circulation, and epigenetic mechanisms following environmental exposures. Further studies should unravel the subtle and fundamental pathophysiological mechanisms explaining the myriad effects of GPBAR-1, mainly to improve liver protection and to facilitate beneficial metabolic effects through primary prevention measures.

## Figures and Tables

**Table 1:** Effects of bile acid (BA) interactions with the Farnesoid X receptor (FXR), G protein-coupled bile acid receptor 1 (GPBAR-1), and sphingosine-1-phosphate receptor 2 (S1PR2).

**Receptor**	**Mechanism(s)**
FXR	- Formation of the FXR heterodimers (RXR, pregnane receptor, peroxisome proliferator-activated receptors (PPAR’s), activation of small heterodimer partner (SHP), the main effector of FXR activation [1] - Modulation of BA synthesis, conjugation, transport, and excretion [1,29] - Protection against BA overload in the liver [30,31], secondary to increased intestinal absorption of BA [32] or to a decreased intestinal availability of BA [33] - Induction of the FXR-FXR responsive element (FXRE) and modulation of Fibroblast growth factor FGF15 (animal)/FGF19 (human) levels (ileal enterocyte) [34,35,36] - Modulation of gene expression of transporters involved in the enterohepatic circulation of BA [37,38,39] - Interaction with gut microbiota [40,41,42]
GPBAR-1	- Modulation of BA synthesis, intestinal biotransformation and uptake [1] - Metabolic effects (insulin secretion and/or sensitivity [43,44], intestinotrophic paracrine actions, modulation of appetite, intestinal mucosal growth, activity of brush border enzymes, effects of gastric emptying and on absorption of nutrients) [3,45,46,47] - Effects on energy homeostasis [1,48,49,50] - Effects on the release of nitric oxide from the endothelium [51,52,53] - Effects on portal blood flow (hepatoprotective effects) [54,55] - Immunomodulatory effects (modulation of proinflammatory cytokine expression) [56,57] - Effects on cholangiocytes cilia, proliferation and apoptosis [58,59,60] - Effects on macrophages (anti-inflammatory effects, reduced secretion of proinflammatory cytokines and chemokines, inhibition of NLR Family Pyrin Domain Containing 3 (NLRP3) inflammasome) [61]
S1PR2	- Effects on vascular smooth muscle cells, permeability and inflammation [62,63] - Modulation of BA induced hepatocyte apoptosis [64] - Modulation of gene expression in hepatocytes [62,63] - Modulation of sterol and lipid metabolism in the liver [62,63] - Effects on liver sinusoidal endothelial cells, vascular permeability, inflammation phenotype, cell adhesion [62,63,65,66] - Modulation of fibrogenic gene expression, cell contraction, proliferation and migration in hepatic stellate cells [67] - Effects on cholangiocytes (cell proliferation, migration and invasiveness) [62,63,68] - Modulation of proinflammatory gene expression and inhibition of monocyte adhesion in liver sinusoidal endothelial cells [62,63,69] - Effects on macrophages (proinflammatory effects, secretion of cytokines, enhancement of migration, suppression of phagocytosis) [62,63,69,70]

**Table 2:** Potential mechanisms linking G protein-coupled bile acid receptor 1 (GPBAR-1) to bile acid (BA) pool composition [3,46].

Site/Pathway	Mechanism(s)
**Liver** **(BA synthesis)**	- Control of synthetic pathway [102] - GPBAR-1 KO (knockout) mice → decreased expression of Cyp7b1 gene (reduction of the alternative BA synthesis) → BA metabolism shifts towards the classic pathway→ assembly of more hydrophobic pool (also hepatic growth hormone-signal transducer and activator of transcription 5, Growth hormone (GH)/STAT5 signalling) [102]
**Intestine** **(BA biotransformation)**	- Activation of intestinal farnesoid X receptor (FXR) (e.g., FXR agonist fexaramine) might shape gut microbiota → increase of taurolitocholic acid (TLCA) → activation of intestinal GPBAR-1→ release of Glucagon-like peptide 1 (GLP-1) → improved hepatic glucose and insulin sensitivity and increased adipose tissue browning [91,92,103]
**Enterohepatic cycle, ileum, biliary tract, kidney/transepithelial flux of BA**	- Largely unknown - Regulation of BA transport in the gallbladder epithelial cells (namely, cholecysto-hepatic shunt) → decreased intestinal circulation of BA→ decreased biotransformation of primary to secondary BA → decreased hydrophobicity of the BA pool [104,105]

**Table 3:** Additional effects of G protein-coupled bile acid receptor 1 (GPBAR-1) on endothelium and portal tract.

Site	Mechanism(s)
**Endothelium**	Nitric oxide release → effect on sinusoidal blood flow → hepatoprotective effect [51,52,53]
**Portal tract** **(liver sinusoidal endothelial cells)**	Modulation of portal pressure and flow [55] Regulation of expression/activity of cystathionine-gamma-lipase (CSE), an enzyme essential to the generation of hydrogen sulphide (H2S), a vasodilatory agent. [54] Increased activity of endothelial nitric oxide synthase (eNOS) → release of nitric oxide in liver sinusoidal endothelial cells (LSEC) [51] Inhibition of Endothelin 1 (ET-1) transcription [55]

**Table 4:** Putative mechanisms linking G protein-coupled bile acid receptor 1 (GPBAR-1) activation with anti-inflammatory effects.

Site	Mechanism
Kupffer cells (rat) Macrophages (mouse)	GPBAR-1 activation → decreased LPS-induced cytokine gene induction [77,85]
Macrophages (mouse)	GPBAR-1 activation → suppression of IkBα phosphorylation and p65 nuclear translocation in a cAMP-dependent manner, and NF-κB DNA binding activity and its transcription activity [85]
Macrophages (mouse)	GPBAR-1 activation → reduced chemokine secretion and macrophage migration. [184] Induction of CEBP-β as well as AKT-mTOR complex 1 pathway [184] Potential role in the treatment of insulin resistance and type 2 diabetes mellitus, associated inflammatory, and metabolic disorders [184]
Macrophages (mouse) Mice	Interaction of excess CDCA with GPBAR-1/EGFR downstream signalling and NLRP3 inflammasome [178,179] BA and GPBAR-1 activation → inhibition of NLRP3 inflammasome activation via cAMP-PKA axis. [178,179]

**Table 5:** Toxic products of environmental origin influencing GPBAR-1 gene expression.

Toxic of Environmental Origin	Effect	Model	References
Brominated flame retardants (polybrominated diphenyl ethers, PBDEs) Hexabromocyclododecane (HBCDD)	Increased expression of GPBAR-1 mRNA	Sprague-Dawley rats, ovary	[194]
Bisphenol A (BPA)	Decreased methylation of GPBAR-1 gene	Sprague-Dawley rats, mammary gland	[195]
Bisphenol A (BPA)	Upregulation of GPBAR-1	Sprague-Dawley rats, seminiferous tubule	[196]
Bisphenol A (BPA), F (BPF), S (BPS)	Altered coding and noncoding RNA profiles; increased expression of GPBAR-1 mRNA	Humans, human primary adipocyte	[197]
Elemental carbon (EC) Organic carbon (OC)	Increased expression of GPBAR-1 mRNA	Humans, whole blood	[198]

**Table 6:** Potential drawbacks associated with GPBAR-1 activation.

Site	Mechanism(s)/Evidence	Model(s)
Gallbladder	Increased refilling (relaxation of smooth muscle cells) [100] Gallstone formation (likely due to increased gallbladder stasis and cholesterol accumulation in the gallbladder) [101] Targeted deletion of Gpbar-1 protects mice from cholesterol gallstone formation [101]	Animal
Pancreas	GPBAR-1 mediated pancreatic damage (pancreatitis) [221]	Animal
Peptidergic neurons of mouse dorsal root ganglia and spinal cord Dermal macrophages that contain opioids	Pruritus [222] Analgesia [222] Likely mediated by bile acid activation of GPBAR-1 at specific sites [222] Effects attenuated in GPBAR-1 KO mice and exacerbated in GPBAR-1-Tg mice (overexpressing mouse GPBAR-1) [222]	Animal
Endothelial cells	Reduced blood pressure with GPBA-1 agonists [220] Activation of GPBAR-1 in endothelial cells [220]	Animal
Cholangiocytes	Increased proliferation [58] Reduced apoptosis [58] Tumorigenesis (cholangiocarcinoma) [58]	Animal
Oesophagus	GPBAR-1 highly expressed in oesophageal adenocarcinoma and precancerous lesions (histology) [223] GPBAR-1 stimulation: Increased proliferation in oesophageal adenocarcinoma cells and gastric cancer cells [82,188]	Human (in vivo, in vitro)
